# Effects of Mechanical Stress on Insulation Structure and Performance of HV Cable

**DOI:** 10.3390/polym14142927

**Published:** 2022-07-20

**Authors:** Jingang Su, Liqiang Wei, Jingquan Zheng, Jiahao Liu, Peng Zhang, Xianhai Pang, Yunqi Xing

**Affiliations:** 1 Electric Power Research Institute of State Grid Hebei Electric Power Supply Co., Ltd., Shijiazhuang 050021, China; lw15511735570@126.com (L.W.); fzaj570795@163.com (P.Z.); sdlkzjq123@outlook.com (X.P.); 2 State Key Laboratory of Reliability and Intelligence of Electrical Equipment, Hebei University of Technology, Tianjin 300123, China; jqzhebut@163.com (J.Z.); ljh1484043663@163.com (J.L.); yqxing@hebut.edu.cn (Y.X.)

**Keywords:** polyethylene, mechanical property, molecular dynamics simulation, insulation properties

## Abstract

Mechanical stresses generated during manufacturing and laying process of high voltage cables can result in degradation of insulation properties, affecting the stable operation of the transmission system. Traditional test methods for testing the effect of mechanical stress on the insulation properties of polyethylene still have some shortcomings to be explored and it is able to explain the changes of the insulation properties of polyethylene under mechanical stress from a microscopic perspective. In order to further study the effect of stress on the insulation properties of polyethylene, microstructural changes, the breakdown field strength, conductivity and charge distribution of polyethylene at different elongation rates are investigated by a combination of experimental and molecular dynamics simulations. The results show that the increase in stress leads to a decrease in crystallinity and microcrystalline size of the material decrease. The untwisting and orientation of the polyethylene molecular chains during the stretching process can create cavities, resulting in an uneven sample distribution and thickness reduction, leading to a reduction in the breakdown field strength. Meanwhile, some crystal regions are transformed into amorphous regions. The loose amorphous regions facilitate the directional migration of carriers, resulting in the increase of conductivity. When the elongation ratio is smaller, the distance between the molecular chains increases and the trap depth of the specimen becomes shallower. This facilitates the migration of ions and electrons and increases the rate of decay of the surface potential. When the stretch is further increased, new traps are created by broken molecular chains to limit the movement of charges, decreasing the decay rate of the surface potential and reducing the insulation properties of the polyethylene. Meanwhile, the molecular dynamics model of semi-crystalline polyethylene was developed to observe the microstructure and energy changes during the stretching process. The conclusions in terms of tensile tests were verified from a microscopic perspective.

## 1. Introduction

High-voltage cables occupy a very important position in the urban electricity supply system with the scale of China’s urban electricity supply continues to expand [1]. Polyethylene cables with excellent electrical properties are now widely used in power transmission lines [2,3]. However, polyethylene insulation is subject to stress concentrations in certain areas during production, installation and operation due to the bending process required, this phenomenon leads to a decrease in insulation performance [4,5]. The distribution of surface charge is strongly related to the energy levels of the local states generated by defects in the material itself, yet conventional test methods have difficulty in explaining the evolution of polyethylene insulation properties in relation to changes in the structure and energy levels of the aggregated states from a microscopic perspective. This paper therefore uses a combination of experimental and molecular dynamics methods to investigate the effect of mechanical stress on the aggregated state and insulation properties of polyethylene, explain the effect of changes in material structure on the electrical properties of the material from a microscopic perspective and provide some reference for stress control in high voltage cables.

Many researchers have carried out studies on the morphological structure changes and trap distribution of cable insulation materials due to mechanical stress. Wang et al. investigated the relationship between space charge and morphological structure of low-density polyethylene and showed that the increase of crystallinity and the decrease of spherical crystal size would reduce the accumulation of space charge [6]. E. David et al. found that a smaller tensile strain increases the breakdown strength of polyethylene materials [7,8]. A. I. Mohamed et al. investigated the effect of compressive stress on the space charge properties of low density polyethylene. The results of the study showed that the penetration of space charge within the sheet was limited when the material was subjected to compressive stress [9]. Du et al. measured the trap distribution of PP/POE blends with different elongation ratios and found that the trap depth first became shallower and then increased as the elongation ratio increased, showing that the main reason why stretching affects the trap distribution characteristics is the change in specimen structure [10]. In contrast to traditional experimental testing methods, molecular dynamics simulations allow the analysis of material properties at the microscopic scale. Molecular dynamics simulations could explain the mechanisms influencing the macroscopic properties to reduce the cost of testing and shorten the research period. Make et al. performed uniaxial and multiaxial molecular dynamics tensile testing simulations of polymers, the results show that the processes under uniaxial and multiaxial stretching are not affected by the setting of boundary conditions [11]. Chen et al. carried out molecular dynamics simulation of graphene/polyethylene composites under uniaxial tension to obtain the variation of the stress-strain curve at different tensile rates [12]. The mechanical properties were studied when the semi-crystalline polyethylene model was subjected to three stresses in compression, tension and shear. The results of the study show that wafer slippage occurs in the elastic phase when subjected to tensile action [13,14,15,16,17]. Queyroy et al. investigated the tensile process of semi-crystalline polyethylene and showed that the yield limit is related to the thickness of the crystalline zone [18]. Yeh et al. investigated the effect of system size on the stretching process of semicrystalline polyethylene by means of molecular dynamics simulations which showed that the system size had little effect on the stretching process [19]. Most of the current studies on the effects of stress on polyethylene insulation properties in high-voltage cables have been explained experimentally. However, a mechanistic explanation of material microstructure changes on breakdown field strength, conductivity and surface charge accumulation is lacking.

The crystallinity and microcrystal size of polyethylene at different elongation ratios was tested by x-ray diffractometer. The breakdown field strength, electrical conductivity and space charge distribution of polyethylene specimens were tested under different elongation ratio conditions. The polyethylene model was constructed with mixed crystalline and amorphous regions and the structural change diagrams, stress-strain curves, potential energy change curves and energy band structure diagrams of the stretching process were recorded to explain the insulation properties of polyethylene at different elongation ratios in terms of slipping, untwisting and orientating of the molecular chains.

## 2. Materials and Methods

### 2.1. Specimen Preparation and Test Set-Up

In this paper, test samples were prepared using a high-density polyethylene material. The raw materials were placed in an open refiner at an experimental temperature of 120 °C for 15 min of melt blending, followed by hot pressing on a flat vulcanizing machine at a temperature of 115 °C and a pressure of 15 MPa for 15 min to prepare 40 × 40 × 0.4 mm^3^. λ was the tensile ratios of specimens, λ was set to 0%, 10%, 20% and 30% by using a tensile machine at a rate of 5 mm/min.

**Figure 1:**
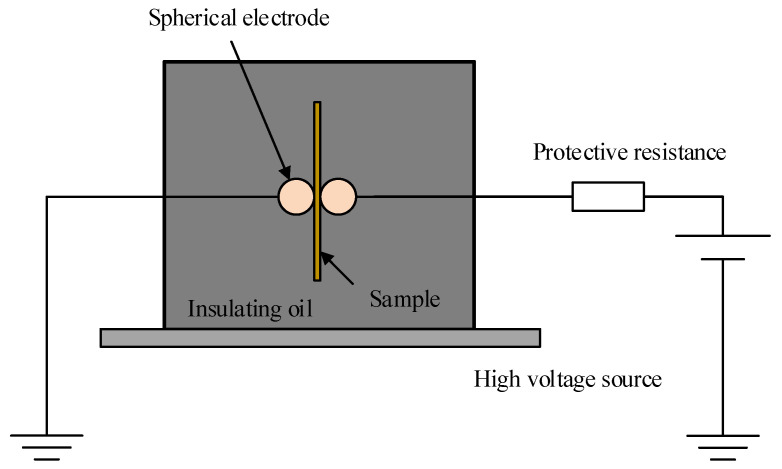
Breakdown field strength test device.

According to national standard GB/T 1408.1-2006, the relationship between the tensile stress and the insulation properties of the material was investigated by subjecting four groups of polyethylene specimens with different elongation ratios to a frequency AC breakdown test. The experimental electrode is a spherical electrode with a diameter of 25 mm, and the polyethylene breakdown experimental setup is shown in Figure 1 [20,21]. The voltage is increased to 15 kV and then the voltage is increased at a constant rate until the sample breaks down. Two parameter Weibull distribution was used to record the data of 10 breakdown experiments of each group of samples. The characteristic breakdown field strength of the material was expressed as the breakdown field strength corresponding to a cumulative failure probability of 63.2% [22,23].

**Figure 2:**
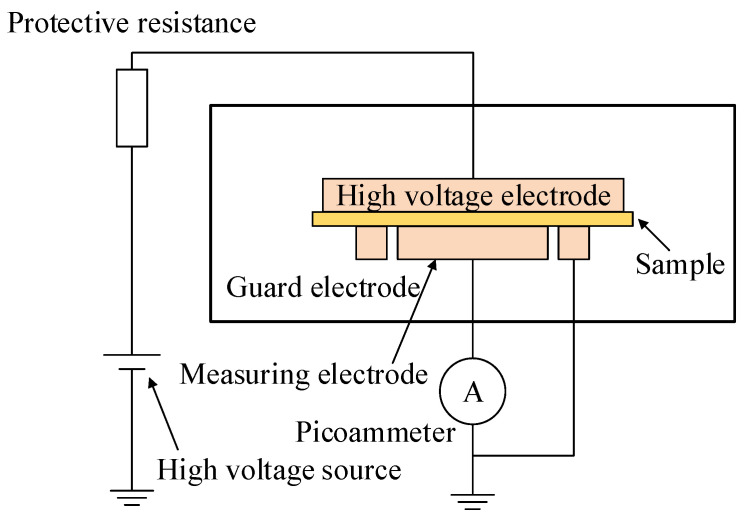
Schematic diagram of DC conductivity test.

According to national standard GB/T 31838.3-2019, the straight fluid conductivity was measured at different elongation ratios using the three-electrode method [24]. The measurement device is shown in Figure 2 and the current value for each specimen under the condition of keeping the voltage stable for 10 min is used as the value of body conductivity current [25]. Each sample was tested six times and averaged to calculate the conductivity of the material.

**Figure 3:**
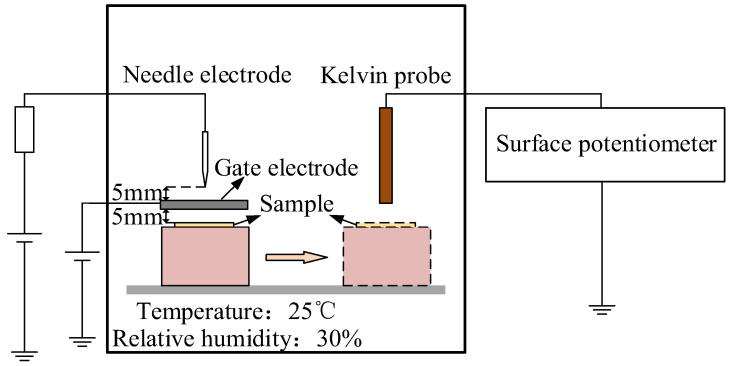
Schematic diagram of corona and surface potential measurement system.

Figure 3 shows the constructed experimental test platform. In this paper, the isothermal surface potential method is used to measure the trap energy level distribution characteristics of polyethylene specimens at different elongation ratios.

Charging of polyethylene surfaces was with the needle-gate electrodes. The needle electrode is ramped up to 5 kV and the gate electrode is ramped up to 2.5 kV at a constant rate and charged for 20 min during the test. After charging, turn off the power immediately and move the specimen to the Kelvin probe below quickly, the change pattern of the material surface potential is recorded by the electrostatic meter and the probe is aligned with the charging position during the recording process [26]. The external environmental factors were kept with the relative humidity of 30% and the constant temperature of 25 °C.

### 2.2. Simulation Model Construction

**Figure 4:**
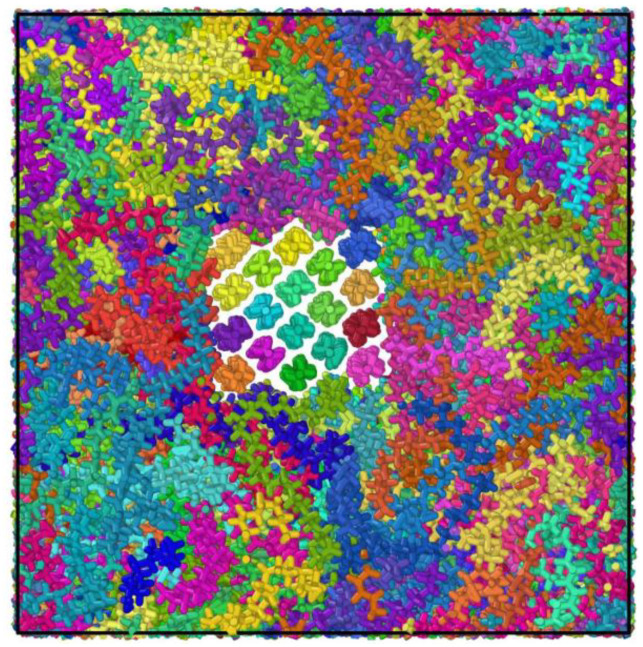
Molecular dynamics modeling of the mixture polyethylene structures.

The polyethylene model with mixed crystalline and amorphous regions was built from the view of microscopic or submicroscopic. Preliminary model was with 4 × 4 × 80 crystalline and 80 irregular molecular chains of 100-monomer, and the initial amorphous model density is set to 0.8 g/cm^3^ [27,28]. In the simulation, it was subjected to 0.5 × 10^4^ fs of constant pressure relaxation under a regular system synthesis (NVT) with reaction conditions set to 500 K with the time step of 0.5 fs, followed by 0.5 × 10^4^ fs of constant pressure relaxation under an isothermal isobaric system synthesis (NPT) with reaction conditions set to 500 K with the time step of 0.5 fs. The microscopic model of the polyethylene equilibrium state is shown in Figure 4. The semi-crystalline polyethylene system was then subjected to uniaxial stretching simulations at a constant rate with a zero-pressure condition for the two lateral simulation cell faces. The semi-crystalline polyethylene configurations were uniaxially deformed at a strain rate of 10^7^ s^−1^.

## 3. Results and Discussions

### 3.1. Effect of Stress on the Insulation Properties of Polyethylene

**Figure 5:**
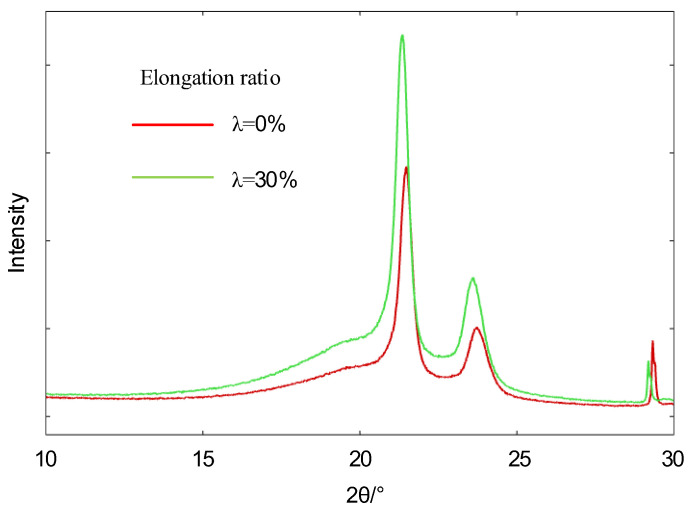
XRD patterns of polyethylene with different elongation ratios.

Figure 5 shows the X-ray diffractograms of polyethylene at different stretching ratios. The polyethylene has high diffraction intensity (110) crystalline diffraction peaks and (200) crystalline diffraction peaks near 21.35° and 23.60°. The crystallinity of the measured samples was measured by fitting the curves in the obtained spectra to the split peaks using the Gauss-Cauchy method and calculating the integration of the fitted curves. The calculation formula is shown in Equation (1) [29].

(1) $$W=\frac{I_{\left(110\right)}+1.42I_{\left(200\right)}}{I_{\left(110\right)}+1.42I_{\left(200\right)}+0.68I_{A}}\times 100\%$$

Using the Scherrer formula shown in Equation (2), the size of the microcrystal perpendicular to the crystal plane L can be found as follows [30]:

(2) $$L=\frac{0.89\lambda }{\beta cos\theta }=\frac{0.89\lambda }{\sqrt{B^{2}-b^{2}}cos\theta }$$

Table 1 shows the XRD parameters of polyethylene at different stretching rates. The crystallinity of the material decreases and microcrystalline size of the material decrease due to the increase in tensile stress. The results are related with the break up of crystalline in polyethylene.

**Figure 6:**
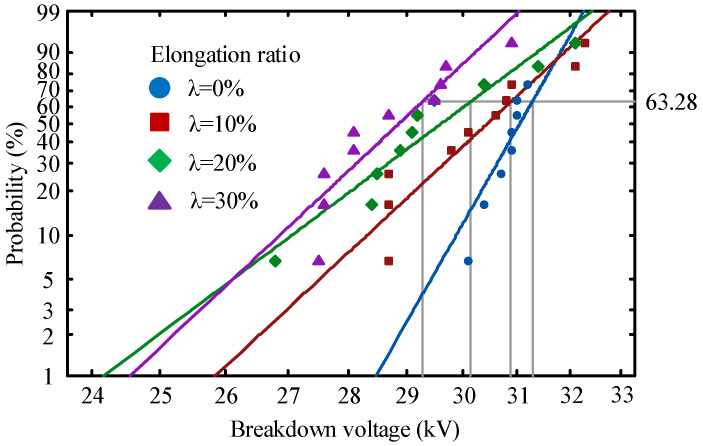
AC breakdown voltage of polyethylene at different elongation ratios.

Figure 6 shows the AC breakdown voltage distribution of polyethylene at different elongation ratio. The breakdown voltages of the four samples at 63.28% breakdown probability are 31.25 kV, 30.59 kV, 30.22 kV and 29.18 kV. Table 2 shows the Weibull parameters for the breakdown voltage of polyethylene at different elongation ratios. The decrease in the shape parameter indicates a greater dispersion of the breakdown test results [31]. In this paper, the breakdown field dispersion of original polyethylene is the largest and the breakdown voltage is the highest because of its most uniform internal structure. The degree of defects in different places inside the stretched polyethylene leads to the generation of electrical dendrites that accelerate insulation failure [32]. This phenomenon leads to a decrease in shape parameters and breakdown voltage.

**Figure 7:**
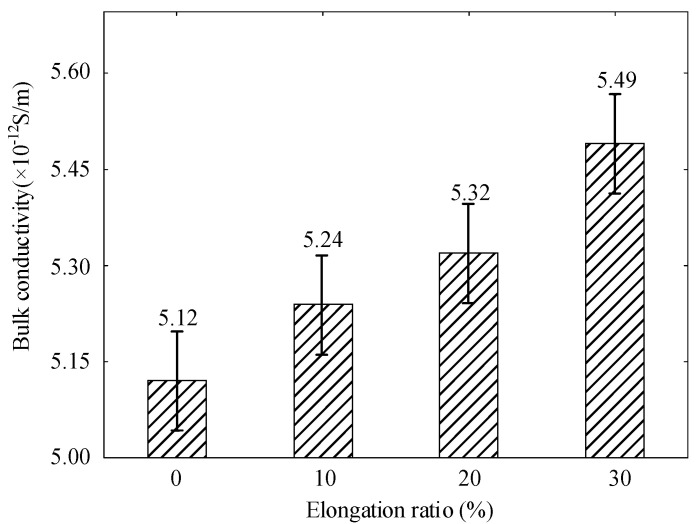
Electrical conductivity of polyethylene at different elongation ratios.

Figure 7 shows the electrical conductivity of polyethylene at different elongation ratios. According to the theory of free volume, there is some free volume between the crystalline and amorphous regions of polyethylene and the free volume increases under the action of stretching. The migration rate of carriers under the action of electric field increases [33]. The conductivity of polyethylene gradually increases as the elongation ratio increases. The tensile stress on the specimen also becomes larger when the elongation ratio increases. Some amorphous zones are not converted to crystalline zones in time. Accompanied by slippage between the tensile grains of the specimen to result an increase in the proportion of amorphous regions and the release of part of the carriers by traps [34]. As the stretching process increases, the crystallinity decreases with the free volume in-creasing inside the polyethylene, According to the free volume theory, the increased free volume results to an increase in carrier mobility [35].

**Figure 8:**
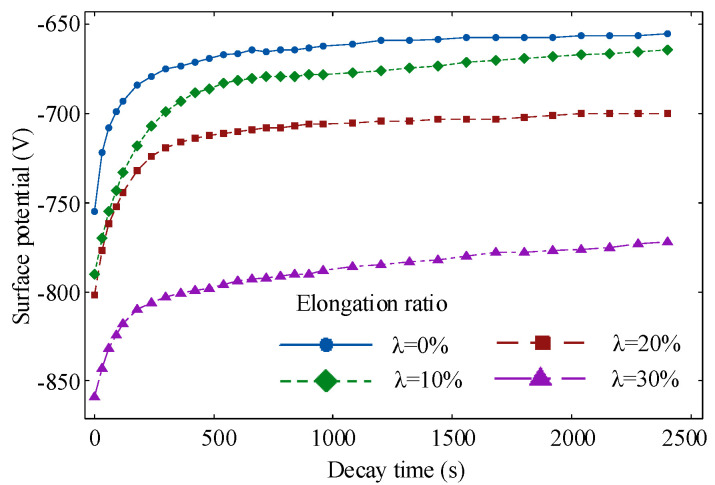
Decay characteristics of polyethylene surface potential at different elongation ratios.

**Figure 9:**
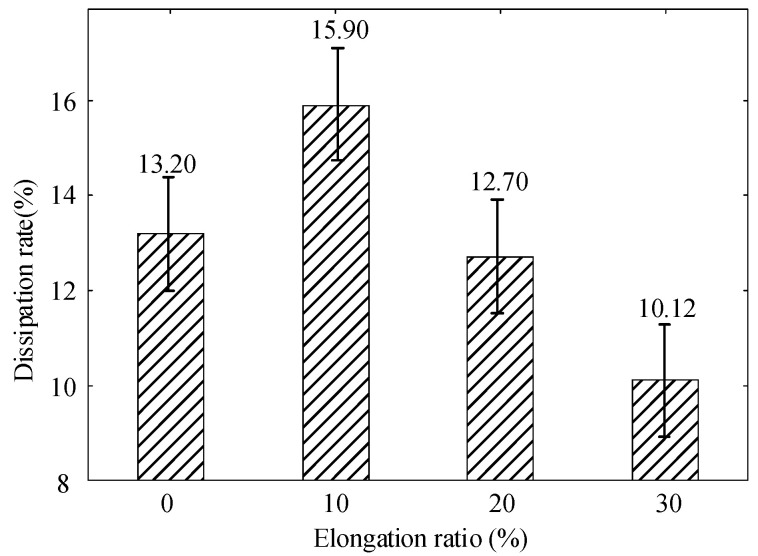
Decay rate of polyethylene surface potential at different elongation ratios.

Figure 8 and Figure 9 show the decay characteristics of the polyethylene surface potential at different elongation ratios. The decay rate of the surface potential increases when the elongation ratio is 10%, it decreases when the elongation ratio is 20% or 30%. It reaches a maximum of 15.90% at a elongation ratio of 10% and a minimum decay rate of 10.12% at a elongation ratio of 30%. The internal trap characteristics of the material are a key factor in the rate of decay.

In this paper, the isothermal surface potential decay method is used to analyze the trap distribution characteristics of polyethylene at different elongation ratios. According to the isothermal surface potential decay model proposed by Simmons [36], the relationship between surface trap energy level and trap density can be derived as:

(3) $$\frac{N_{t}\left(E\right)}{E_{t}}=\frac{4\varepsilon_{0}\varepsilon_{r}}{eL^{2}k^{2}T^{2}ln\left(vt\right)}\left|t\frac{dV\left(t\right)}{dt}\right|$$

**Figure 10:**
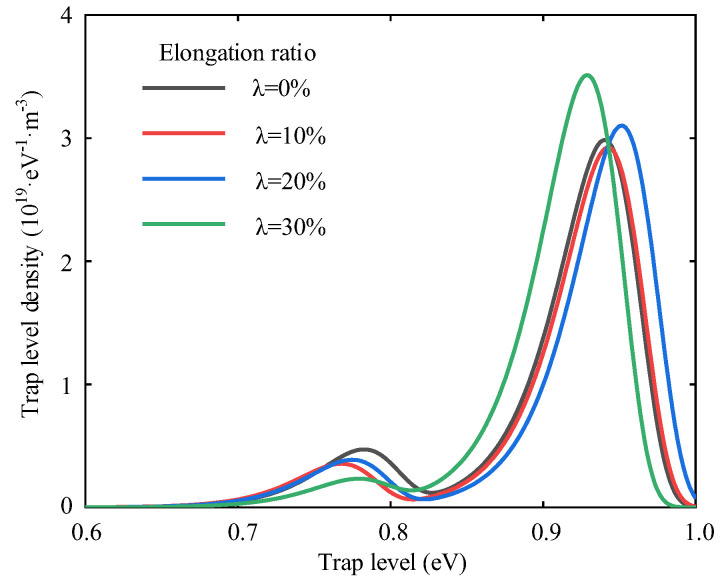
Energy level distribution of polyethylene trap at different elongation ratios.

When the polyethylene is subject to tensile stress, the interface between the crystalline and amorphous zones is destroyed, which leads to the differences of trap distribution [37]. The trap energy level distribution of polyethylene at different elongation ratios is shown in Figure 10. Negative ions and electrons are stacked inside the specimen. The variation pattern of the trap energy level shows a trend of decreasing and then increasing with the increasing elongation ratio. When the polyethylene elongation ratio is 10%, the internal molecular chains of polymers are subject to stresses that cause orientation and untwisting movements and the distance between molecular chains will increase. This increase will facilitate the migration of ions and electrons in the molecular chain. The trap depth of the specimen becomes shallow and the surface potential decay rate becomes large [38]. The untwisting and orientation between the molecular chains contribute to the decay of the surface potential when the stretching is relatively small. The distance between the molecular chains increases further and even causes the molecular chains to break when the polyethylene elongation ratio increases to 30%. The broken molecular chains create new traps that limit the movement of charges. This results in charge accumulation and a decrease in the decay rate of the surface potential, which can reduce the insulation properties of polyethylene.

### 3.2. Effect of Stress on the Microstructure of Polyethylene

**Figure 11:**
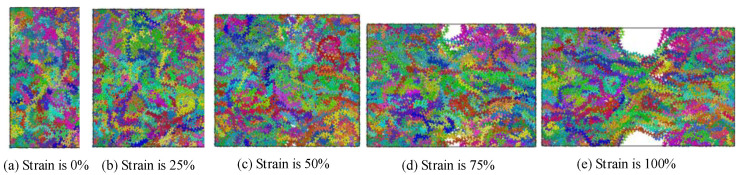
Tensile process of the mixture polyethylene structure.

**Figure 12:**
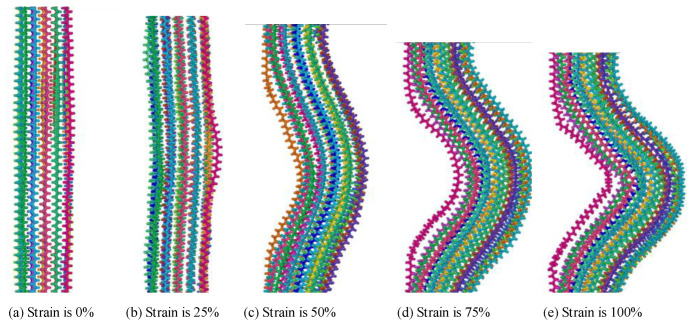
Tensile process of crystalline in the mixture polyethylene structure.

The polyethylene model is made up of a mixture of crystalline and amorphous zones. A periodic boundary condition is employed in this paper. A semi-wafer lamination model was prepared to study the tensile process [39]. The structural changes of polyethylene during the tensile process are shown in Figure 11 and Figure 12. During the tensile process, the deformation of the amorphous region of polyethylene will occur under the action of the force. The untwisting and orientation of the chains leads to the formation of small cavities which can extend through the middle of the system and eventually lead to large cracks or even fracture of the whole system [40], thus affecting the properties of polyethylene. The essence of the deformation of the amorphous region is the mutual restriction and coordination of the entanglement morphology changes at different scales.

In the process of stretching, the volume of the system gradually increases and the molecular chain moves in the direction of stress due to the action of the force. The whole crystal domain still maintains the parallel arrangement structure of folded chain, but some chain segments in the crystal domain slip, resulting in the decrease of the tightness of the arrangement between the chain segments and leading to the slip of the crystal region [41]. With the continuous increase of strain in the tensile process, the crystalline region slides, part of the crystalline region changes to amorphous region. 

**Figure 13:**
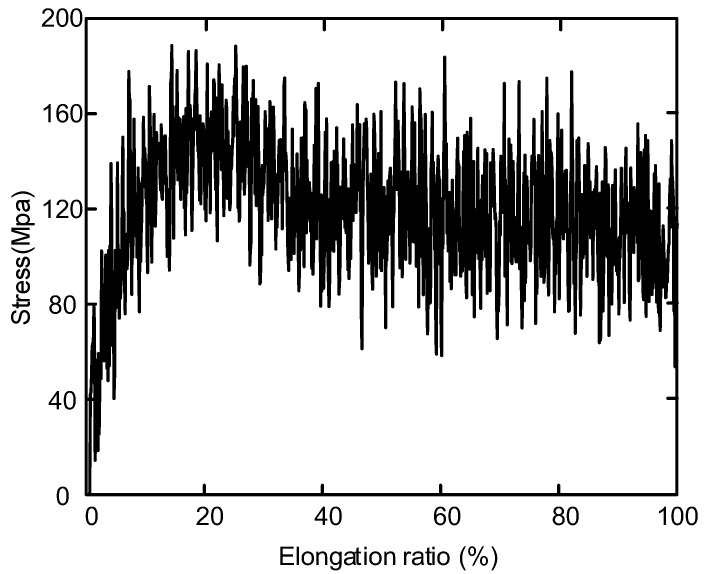
Stress-strain response of the mixture polyethylene structure deformed in uniaxial tension.

Figure 13 shows the variation of stress and strain in uniaxial tensile simulation of polyethylene. Internal stress relaxation and thermal equilibrium of the system during tensile simulation can lead to stress oscillations. In the range of 0 < strain ≤ 20%, the stress increases rapidly with the increase of strain. This stage is elastic deformation stage, indicating that the material is elastic. When the strain is about 20%, the yield limit is reached. When 30% < strain ≤ 60%, the stress decreases with the increase of strain, and this stage is stress softening stage. When strain > 60%, stress increases continuously.

**Figure 14:**
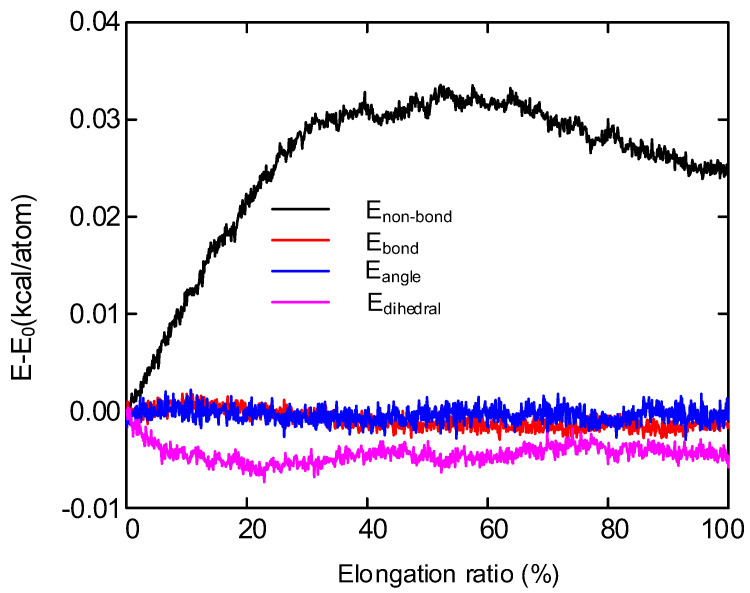
Energy decomposition for the mixture polyethylene structure.

Figure 14 shows the variation of non-bond energy, bond energy, bond angle energy and dihedral energy of polyethylene during tensile process. Initially, the molecular chains are tightly pressed together. With the increase of strain, the orientation and relative movement of polyethylene molecular chains occur in the tensile process, and the free volume between adjacent molecules increases, resulting in the rapid rise of non-bond energy [42]. After reaching the yield limit, the non-bond energy is stable and increases slowly with the further increase of strain. In addition, the bond energy, bond angle energy and dihedral en-ergy of polyethylene molecules have almost no change in the tensile process.

**Figure 15:**
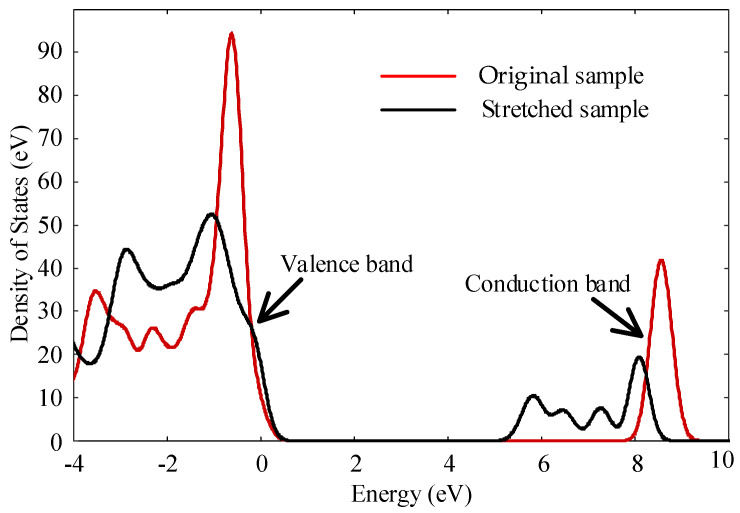
Density of states diagram before and after stretching.

Figure 15 shows the density of states of polyethylene single chain before and after stretching calculated by Dmol3 module. The program was written by MS perl language script, and 5 Gpa force was applied for uniaxial tension. The band gap width of polyethylene single chain before stretching is about 6.6 eV, which is similar to the existing studies [43]. The peak values of the density of states of the stretched polyethylene decrease significantly at the valence band and conduction band, and a new peak appears at the conduction band, resulting in the reduction of the band gap to 3.8 eV. In this paper, it is considered that stretching will lead to deformation of macromolecules and affect the structure of aggregated states, thus introducing new local states in polyethylene materials. Therefore, tensile treatment will introduce a large number of charge traps into the material, resulting in an increase in the number of traps, which will affect the properties of polyethylene [44].

## 4. Conclusions

In this paper, a combination of experimental and molecular dynamics methods was used to observe the microstructural changes of polyethylene at different elongation rates and combined with the changes of energy and microstructure of semi crystalline polyethylene during uniaxial tension to explain the changes in breakdown field strength, conductivity and space charge properties. The following conclusions are obtained in the paper.

1. The crystallinity and microcrystalline size of polyethylene decreased at a stretching ratio of 30% compared to unstretched. It is due to the increase of tensile stress which makes the grain breakage and leads to the obstruction of the orientation movement of molecular chains.
2. With the increase of tensile stress, the breakdown field strength of polyethylene decreases continuously. At the same time, conductivity increases with the increase of stretching. When the elongation ratio is 10%, the distance between the molecular chains becomes larger, which is conducive to the migration of ions and electrons, and the decay rate of surface potential increases. As the stretching increases further, more defects are created within the sample, the trap depth becomes deeper, which reduces the rate of decay of the surface potential, resulting in a large amount of charge accumulation in the sample and reduces the insulation performance of the material.
3. The main structural changes of semi crystalline polyethylene during stretching are the orientation and unwrapping movement of molecular chain, the sliding of crystal region and the transformation of part of crystal region to amorphous region. The simulated stress-strain curve includes four parts: elastic deformation, yield, strain softening and strain strengthening. Non-bonding energy play an important role in the potential energy change during stretching. The changes in the microstructure of polyethylene during the experimental process are verified by simulating the motion states of the molecules observed during the stretching process. The changes in the electrical properties of polyethylene during the stretching process are explained from a microscopic point of view.

## Figures and Tables

**Table 1:** XRD parameters of polyethylene after tensile treatment at different ratios.

Elongation Ratio	L(110)/nm	L(200)/nm	W/%
0%	9.1	9.9	58.1%
30%	8.2	9.6	46.13%

**Table 2:** Weibull parameters of AC breakdown voltage.

Elongation Ratio	Scale Parameters/kV	Shape Parameters	Standard Deviations
0%	31.25	46.29	0.56
10%	30.59	28.04	1.26
20%	30.22	20.56	1.45
30%	29.18	24.60	1.09

## Data Availability

All data generated or analyzed during this study are included in this article.
